# Exploring the Symbiotic Mechanism of a Virus-Mediated Endophytic Fungus in Its Host by Dual Unique Molecular Identifier–RNA Sequencing

**DOI:** 10.1128/mSystems.00814-21

**Published:** 2021-09-14

**Authors:** Zheng Qu, Hongxiang Zhang, Qianqian Wang, Huizhang Zhao, Xiaofan Liu, Yanping Fu, Yang Lin, Jiatao Xie, Jiasen Cheng, Bo Li, Daohong Jiang

**Affiliations:** a State Key Laboratory of Agricultural Microbiology, Huazhong Agricultural University, Hubei Province, Wuhan, China; b Hubei Key Laboratory of Plant Pathology, Huazhong Agricultural University, Hubei Province, Wuhan, China; c Hubei Hongshan Laboratory, Wuhan, China; Oak Ridge National Laboratory

**Keywords:** *Sclerotinia sclerotiorum*, SsHADV-1, dual-UMI RNA-seq, symbiosis

## Abstract

The symbiosis of endophytes and plants is universal in nature. However, how endophytes grow in plants is not entirely clear. Previously, we reported that a virus-infected fungal pathogen could grow in plants as an endophyte. In this study, we utilized Sclerotinia sclerotiorum strain DT-8, a virus-mediated endophyte, to investigate the mechanism of symbiosis with rapeseed by dual unique molecular identifier–RNA sequencing (dual-UMI RNA-seq). We found that the expressions of genes encoding *S. sclerotiorum* amylase/glucoamylase, glucose transporters, and rapeseed sugars will eventually be exported transporter 11 (SWEET11) were upregulated. It suggested that strain DT-8 might utilize plant starch as a nutrient. The defense systems of rapeseed were also activated, such as production of reactive oxygen species, phenylpropanoids, and brassinin, to control the growth of strain DT-8, while strain DT-8 counteracted host suppression by producing effector-like proteins, detoxification enzymes, and antioxidant components. Moreover, rapeseed also upregulated pectate lyase and pectinesterase genes to facilitate the colonization by strain DT-8. Our findings provide novel insights into the interaction of virus-mediated endophytes and their hosts that warrant further study.

**IMPORTANCE** Although endophytes are widespread in nature, the interactions between endophytes and their hosts are still not fully understood. Members of a unique class of endophytes, the virus-mediated endophytic fungi, are continuously being discovered and have received wide attention. In this study, we investigated the interaction between a mycovirus-mediated endophytic fungus and its host rapeseed by using dual-UMI RNA-seq. According to the dual-UMI RNA-seq results, an aerial view of symbiotic mechanism under balanced regulation was suggested. This research expands our understanding of the symbiotic mechanisms of virus-fungus-plant interactions and could establish a foundation for the further development of practical application with virus-mediated hypovirulent fungi.

## INTRODUCTION

All plants in natural ecosystems appear to be symbiotic with fungal endophytes (1). Many endophytic fungi have been reported to aid their host plants in resisting biotic stresses from pathogens and herbivores (2). For example, Tian et al. found that Sclerotinia sclerotiorum, a widespread pathogen of dicotyledons, could grow endophytically in gramineous plants and provide protection against many plant-pathogenic fungi (3). The endophytic fungus of tall fescue (Festuca arundinacea), Neotyphodium coenophialum, produces bioactive alkaloids that aid its plant hosts in avoiding ingestion by herbivores (2, 4). Some beneficial endophytic fungi also could help their host plants resist external abiotic stresses, such as salinity, heavy metal, and drought stresses (5). Under salinity stress, an endophytic isolate of the fungus Yarrowia lipolytica promoted the growth of maize through metabolism regulation and hormonal secretions (abscisic acid and indoleacetic acid) (6). The root-endophytic fungus of maize, Exophiala pisciphila, could enhance the tolerance of maize to Cd (7). The endophytic fungus Beauveria bassiana could enhance drought tolerance in red oak seedlings by promoting root growth (8). Moreover, endophytic fungi are also important sources of secondary metabolites and bioactive compounds with promising applications in agriculture, therapeutics, and industry (5).

The plant immune system is effective against several types of attackers. Endophytes can enhance plant immunity to respond to abiotic stress or biotic stresses by producing protective metabolites and modulating phytohormone pathways (2). However, the first challenge for endophytes interacting with live plant partners is how to deal with and control plant immunity (9). The intrinsic mechanism for the interaction of endophytic fungi and the plant immune system is complex and remains largely unclear (2). The balanced antagonism hypothesis posits that a balance exists between plant defensive responses to endophytes and the virulence factors of endophytes in plants. With this mechanism, endophytes produce metabolites to overcome the host defense response and succeed in surviving within their hosts. In this way, plant-endophyte interactions are maintained if there is a balanced antagonism between host defense and fungal virulence (10). For example, the taxol produced by a yew-endophytic fungus (Paraconiothyrium SSM001) could resist pathogens, while excessive taxol was harmful to the plant itself by disruption of plant cell cytokinesis (11).

The bidirectional exchange of plant-fixed carbon for fungally acquired nutrients is central to plant-fungal symbioses (12). Fungal symbionts can transfer phosphorus, nitrogen, and micronutrients to their plant hosts. In most cases, fungi provide the host plants with nutrients from the soil (13, 14). The plant typically reciprocates by transferring plant-derived carbohydrates, lipids, or fatty acids to the fungus (12, 15). Although the number of fatty acid fluxes between symbionts and the relative proportions of sucrose/fatty acids transferred between symbionts remain unknown, most models for carbon transport between symbionts are based on the assumption that hexose sugars form the major substrate for exchange (12). However, for many of these fungi, the specific mechanisms and gene products involved in nutrient transfer remain to be elucidated (13).

Mycoviruses or fungal viruses are very common in fungi, including in the endophytic fungi (16, 17). Usually, mycoviruses do not affect the host phenotype (18). However, some mycoviruses could confer special characteristics to their hosts, e.g., defending their fungal hosts from the abiotic stress (19). For example, infection with Curvularia thermal tolerance virus (CThTV) enhanced the heat tolerance of Curvularia protuberata, an endophyte of panic grass. Furthermore, colonization by CThTV-infected C. protuberata can also confer heat tolerance to its plant host (20, 21). A comparative transcriptome study indicated that the presence of CThTV affects the expression of a number of heat stress-related genes in *C. protuberata*, including melanin, many osmoprotectant biosynthetic genes, and heat shock protein genes (21). In addition, to control plant disease, the mycoviruses that modulate endophytic and phytopathogenic fungal traits have attracted a great deal of attention. As a kind of endophytic fungi whose endophytic state is maintained by the infection of mycovirus, virus-mediated endophytes are identified for potential alternative approaches to plant protection and to benefit crop production (22).

As a necrotrophic fungus and the causal pathogens of *Sclerotinia* stem rot (SSR), Sclerotinia sclerotiorum (Lib.) de Bary can cause disease and rapidly kill host rapeseed (Brassica napus) in the greenhouse, and it accounts for huge yield losses in the field (23, 24). Biological control strategies that use mycovirus to control SSR may be an environmentally friendly alternative to reduce the amount of chemical fungicides used (25). In addition to irregular growth, abnormal pigmentation, and hypovirulence, Sclerotinia sclerotiorum hypovirulence-associated DNA virus 1 (SsHADV-1) switches its host, *S. sclerotiorum* strain DT-8, from being a fungal pathogen to being an endophyte in rapeseed (26, 27). Thus, the virus-mediated endophytic strain DT-8 may be a useful biological control agent, acting as a plant vaccine to promote the growth and enhance the SSR resistance of rapeseed by regulating the expression of rapeseed genes involved in defense, hormone signaling, and circadian rhythm pathways (27). Through biopriming treatment, strain DT-8 could control SSR and increase yield in the field, impact the composition and structure of microbial communities and enhance the interaction of microorganisms (28). However, the symbiotic mechanism between strain DT-8 and rapeseed is still unclear.

With the increasing sensitivity of high-throughput RNA sequencing (RNA-seq), dual RNA-seq capturing all classes of coding and noncoding transcripts in both the microorganism and the host has been used to study host-microorganism interactions (29). Although RNA-seq is a powerful tool, sequence-dependent bias and the inaccuracy of PCR amplification become obstacles for further applications. To solve this problem, by labeling each cDNA molecule with a unique molecular identifier (UMI) before library construction, digital RNA-seq or unique molecular identifier (UMI) RNA-seq was created (30). In this study, using dual unique molecular identifier–RNA sequencing (dual-UMI RNA-seq), the symbiotic mechanisms between strain DT-8 and rapeseed were demonstrated.

## RESULTS

### Overview of all RNA-seq data of the plant and fungus.

**FIG 1:**
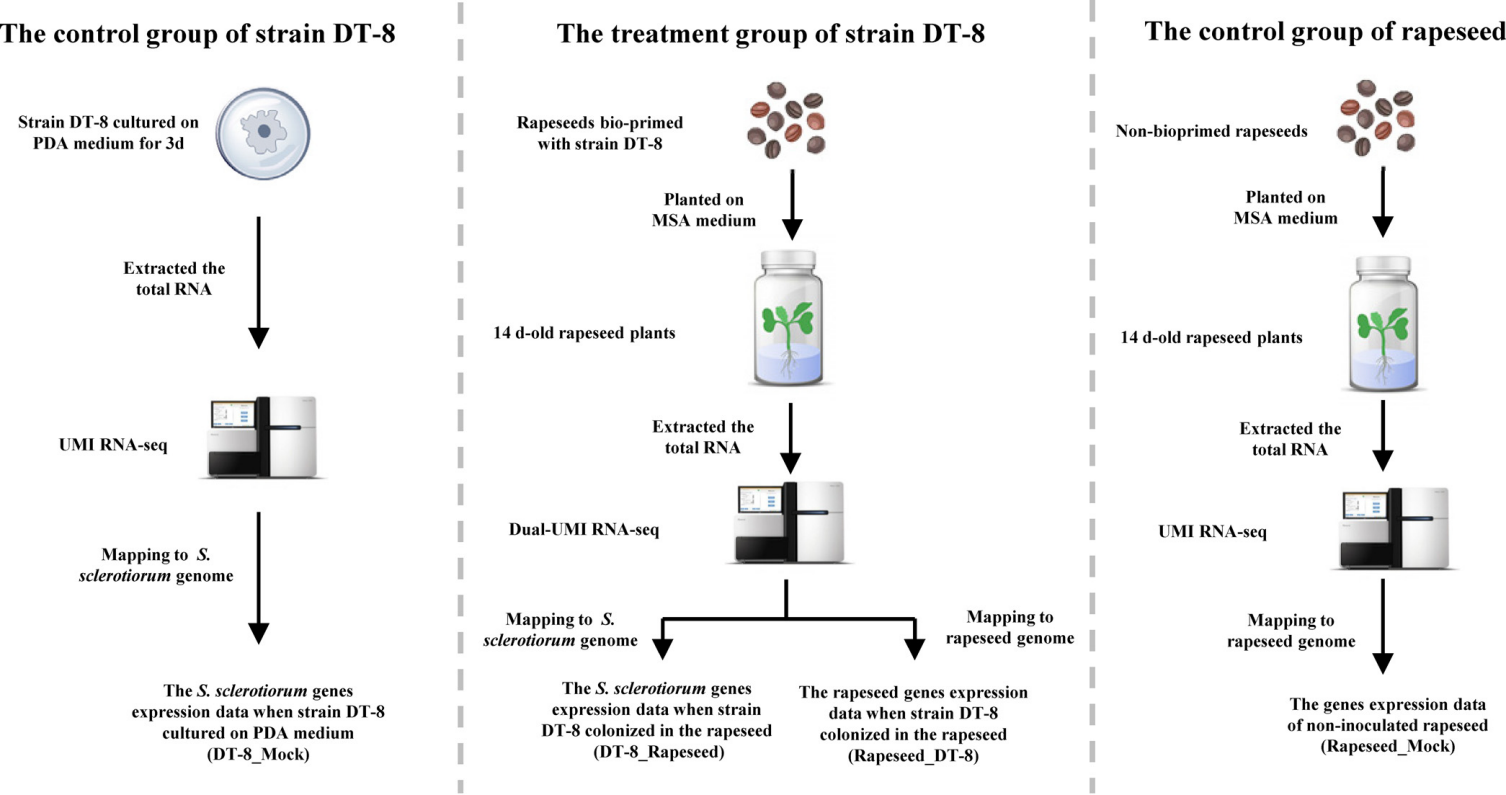
Experimental scheme of the dual unique molecular identifier–RNA sequencing (dual-UMI RNA-seq). For the biopriming treatment, the rapeseeds were primed with strain DT-8 by mixing 5 g of seeds with 10 ml of mycelial suspension for 18 h at 20°C.

To study the symbiotic mechanism between DT-8 and rapeseed, dual-UMI RNA-seq technology was used; the experimental scheme is shown in Fig. 1a. To make strain DT-8 fully colonize in rapeseed, the rapeseeds were bioprimed with mycelial suspension of strain DT-8 for 18 h at 20°C and planted on starch-free Murashige and Skoog agar (MSA) medium in bottles for 14 days at 20°C with 12 h light and 12 h dark.

According to the principal-component analysis (PCA), the three biological triplicates of each group clustered together (see Fig. S1a and b in the supplemental material). In the strain DT-8 treatment group, a total of 207 million reads were obtained in the six samples, of which approximately 4.15% and 83.40% could be aligned to the *S. sclerotiorum* (DT-8_Rapeseed) and rapeseed (Rapeseed_DT-8) genomes, respectively. For the strain DT-8 control group, there were 88 million reads in three samples, and approximately 90.30% of the reads could be aligned to the *S. sclerotiorum* genome (DT-8_Mock). Moreover, for the strain DT-8 treatment and control group, approximately 0.05% and 1.29% of the reads were aligned to the SsHADV-1 genome, respectively. There were 212 million total reads in the three samples of rapeseed control group, and 95.52% of the reads could be aligned to the rapeseed genome (Rapeseed-Mock) (see Data Set S1, tab 1, in the supplemental material).

10.1128/mSystems.00814-21.1 FIG S1 Principal-component analysis (PCA) of RNA sequencing (RNA-seq) data. (a) PCA for rapeseed dual unique molecular identifier–RNA sequencing (dual-UMI RNA-seq) data. (b) PCA for Sclerotinia sclerotiorum dual-UMI RNA-seq data. Download FIG S1, TIF file, 0.8 MB. Copyright © 2021 Qu et al. 2021 Qu et al. https://creativecommons.org/licenses/by/4.0/ This content is distributed under the terms of the Creative Commons Attribution 4.0 International license.

10.1128/mSystems.00814-21.8 DATA SET S1 (Tab 1) Summary of RNA-seq data. (Tab 2) Gene ontology (GO) enrichment analysis of the upregulated genes of rapeseed. (Tab 3) Kyoto Encyclopedia of Genes and Genomes (KEGG) enrichment analysis of the upregulated genes of rapeseed. (Tab 4) Predicted rapeseed pectinesterase and pectate lyase genes. (Tab 5) Predicted key biosynthesis genes of brassinin from indole glucosinolate. (Tab 6) GO enrichment analysis of the differentially expressed genes (DEGs) of *S. sclerotiorum*. (Tab 7) Genes enriched in GO terms of starch binding and glucan 1,4-alpha-glucosidase activity. (Tab 8) Genes enriched in the KEGG pathway of starch and sucrose metabolism. (Tab 9) Predicted *S. sclerotiorum* sugar transporter genes. (Tab 10) Differentially expressed *S. sclerotiorum* genes of predicted secretory proteins (SPs). (Tab 11) qRT-PCR primers used in this study. Download Data Set S1, XLSX file, 0.06 MB. Copyright © 2021 Qu et al. 2021 Qu et al. https://creativecommons.org/licenses/by/4.0/ This content is distributed under the terms of the Creative Commons Attribution 4.0 International license.

### Overall assessment of the expression profiles of *S. sclerotiorum* and rapeseed genes.

In total, 9,323 *S. sclerotiorum* genes and 45,555 rapeseed genes were detected above the detection threshold of 1 count per million (CPM) in at least three biological replicates (31) of a given condition. In this study, an absolute log_2_ fold change (log_2_FC) of >1 and a false-discovery rate (FDR) of <0.05 were used to define differentially expressed genes (DEGs). To study the relative gene expression of *S. sclerotiorum*, gene expression data when strain DT-8 colonized the rapeseed (DT-8_Rapeseed) were compared to those in the DT-8_Mock libraries. A total of 1,921 genes were found to be statistically significant DEGs in the DT-8_Rapeseed libraries, with 1,015 DEGs upregulated and 906 DEGs downregulated. To study the gene expression difference of rapeseed in response to the colonization of strain DT-8, we compared the Rapeseed_DT-8 libraries with the Rapeseed-Mock libraries and identified 1,082 DEGs, of which 1,048 were upregulated and 34 were downregulated DEGs.

### GO and KEGG enrichment analyses for upregulated rapeseed genes.

For the 1,048 upregulated genes in Rapeseed_DT-8, there were 57 significantly enriched gene ontology (GO) terms that were end nodes in the directed acyclic graphs constructed by Biological Directed acyclic graphs Gene Ontology (BiNGO) (see Data Set S1, tab 2, in the supplemental material). Nine enriched GO terms were involved in plant defense, including “chitin catabolic process,” “response to oxidative stress,” “defense response to fungus, incompatible interaction,” “cellular oxidant detoxification,” “systemic acquired resistance,” “defense response by callose deposition in cell wall,” “hydrogen peroxide catabolic process,” “peroxidase activity,” and “tryptophan biosynthetic process.” These results suggested that plant defense was activated and that the disease resistance of rapeseed might be increased after colonization by strain DT-8. These changes are similar to those described in previous research (27). Moreover, “pectin catabolic process” and “cell wall macromolecule catabolic process” terms were also significantly enriched. The upregulated expression of rapeseed genes involved in the catabolic process of plant cell wall components might be beneficial to the colonization by strain DT-8.

For the upregulated rapeseed genes, there were 22 significantly enriched Kyoto Encyclopedia of Genes and Genomes (KEGG) pathways, and “phenylpropanoid biosynthesis” and “glucosinolate biosynthesis” were related to plant defense (see Data Set S1, tab 3, in the supplemental material). These results also showed that the disease resistance of rapeseed was increased after colonization by strain DT-8. There were four upregulated genes belonging to the “pentose and glucuronate interconversions” pathway, with two genes encoding pectinesterase and two encoding pectate lyase (see Data Set S1, tab 4, in the supplemental material). In this research, the upregulated expression of rapeseed pectinesterase and pectate lyase genes might cause cell wall disassembly and promote the colonization by *S. sclerotiorum* DT-8.

### Key brassinin synthesis genes of rapeseed were upregulated in plants colonized by strain DT-8.

Brassica species can produce many indole-sulfur phytoalexins against pathogens, and brassinin occupies a pivotal node in the proposed biosynthesis of indole-sulfur phytoalexins (32). For Brassica rapa, brassinin-associated β-glucosidase (BABG), dithiocarbamate *S*-methyltransferase (DTC-MT), and cytochrome P450 family 71 subfamily CR (CYP71CR) are three key types of enzymes that transform glucosinolate to isothiocyanate and then transform isothiocyanate to brassinin, spirobrassinin, or cyclobrassinin (33). In our analysis, three BABG genes (*BnaC04g03790D*, *BnaC04g49660D*, and *BnaA04g25750D*), four DTC-MT genes (*BnaA02g12540D*, *BnaA07g03510D*, *BnaA07g03730D*, and *BnaCnng41630D*), and six CYP71CR genes (*BnaA03g01790D*, *BnaA10g24610D*, *BnaA10g24620D*, *BnaC03g02320D*, *BnaC09g49530D*, and *BnaCnng60140D*) were identified in the genome of rapeseed (see Data Set S1, tab 5, in the supplemental material). There were four low-expression (<1 CPM) genes (*BnaC09g49530D*, *BnaA03g01790D*, *BnaC03g02320D*, and *BnaA10g24620D*) among those genes. Except for *BnaA04g25750D*, *BnaC04g49660D*, and *BnaCnng41630D*, all of the remaining six genes were upregulated in Rapeseed_DT-8 (see Fig. S2 in the supplemental material). This result suggests that the synthesis of indole-sulfur phytoalexins was activated when strain DT-8 colonized rapeseed.

10.1128/mSystems.00814-21.2 FIG S2 Expression levels of key biosynthesis genes of brassinin. Download FIG S2, TIF file, 2.7 MB. Copyright © 2021 Qu et al. 2021 Qu et al. https://creativecommons.org/licenses/by/4.0/ This content is distributed under the terms of the Creative Commons Attribution 4.0 International license.

### Upregulated expression of rapeseed sugar transporter genes.

In the process of carbohydrate transport between plants and endophytic fungi, plant sugar transporters (STs) play an important role (34). According to Jian et al. and Zhang et al. (35, 36), there were 22 sucrose transporter/sucrose carrier (SUT/SUC) genes, 68 sugars will eventually be exported transporter (SWEET) genes, and 175 monosaccharide transporter (MST) genes in the rapeseed genome. When strain DT-8 colonized rapeseed, there were six upregulated rapeseed ST genes, and none were downregulated; the six upregulated genes in Rapeseed_DT-8 included which one SUC gene (*BnaC06g32880D*), one SWEET11 gene (*BnaA06g16330D*), and four MST genes (*BnaC03g73840D*, *BnaA01g26430D*, *BnaC01g33830D*, and *BnaC05g46630D*) (see Fig. S3 in the supplemental material). These STs might be closely related to the colonization of strain DT-8 in rapeseed.

10.1128/mSystems.00814-21.3 FIG S3 Expression profiles of rapeseed sugar transporter genes. Download FIG S3, TIF file, 2.1 MB. Copyright © 2021 Qu et al. 2021 Qu et al. https://creativecommons.org/licenses/by/4.0/ This content is distributed under the terms of the Creative Commons Attribution 4.0 International license.

### GO enrichment analysis for *S. sclerotiorum* DEGs.

For the 1,015 upregulated genes, there were 29 significantly enriched GO terms that were end nodes in the directed acyclic graphs constructed by BiNGO (see Data Set S1, tab 6, in the supplemental material). Most significantly enriched GO terms were related to plant cell wall catabolism (“pectin catabolic process,” “l-arabinose metabolic process,” “pectinesterase activity,” “alpha-l-arabinofuranosidase activity,” “polygalacturonase activity,” “cellulose binding,” and “endo-1,4-beta-xylanase activity,” “cellulose catabolic process”), and carbohydrate metabolism (“sugar transmembrane transporter activity,” “carbohydrate: proton symporter activity,” “glucan exo-1,3-beta-glucosidase activity,” and “carbohydrate transmembrane transport”), especially starch metabolism (“glucan 1,4-alpha-glucosidase activity” and “starch binding”). In the GO terms “glucan 1,4-alpha-glucosidase activity” and “starch binding,” three (*SS1G_13809*, *SS1G_08135*, and *SS1G_10617*) and four (*SS1G_13809*, *SS1G_08135*, *SS1G_10617*, and *SS1G_09392*) genes were enriched, respectively, and all of the genes encoded glucoamylases or alpha amylases (see Data Set S1, tab 7, in the supplemental material). This result indicated that starch metabolism was altered when strain DT-8 colonized rapeseed and that starch might be an important nutrient for strain DT-8.

The 906 downregulated genes were enriched in the following eight end-node GO terms: “transmembrane transport,” “threonine-type endopeptidase activity,” “integral component of plasma membrane,” “proteasome core complex,” “telomere maintenance,” “DNA repair,” and “nuclear chromosome, telomeric region” (see Data Set S1, tab 6, in the supplemental material).

### KEGG enrichment analysis for *S. sclerotiorum* DEGs.

**FIG 2:**
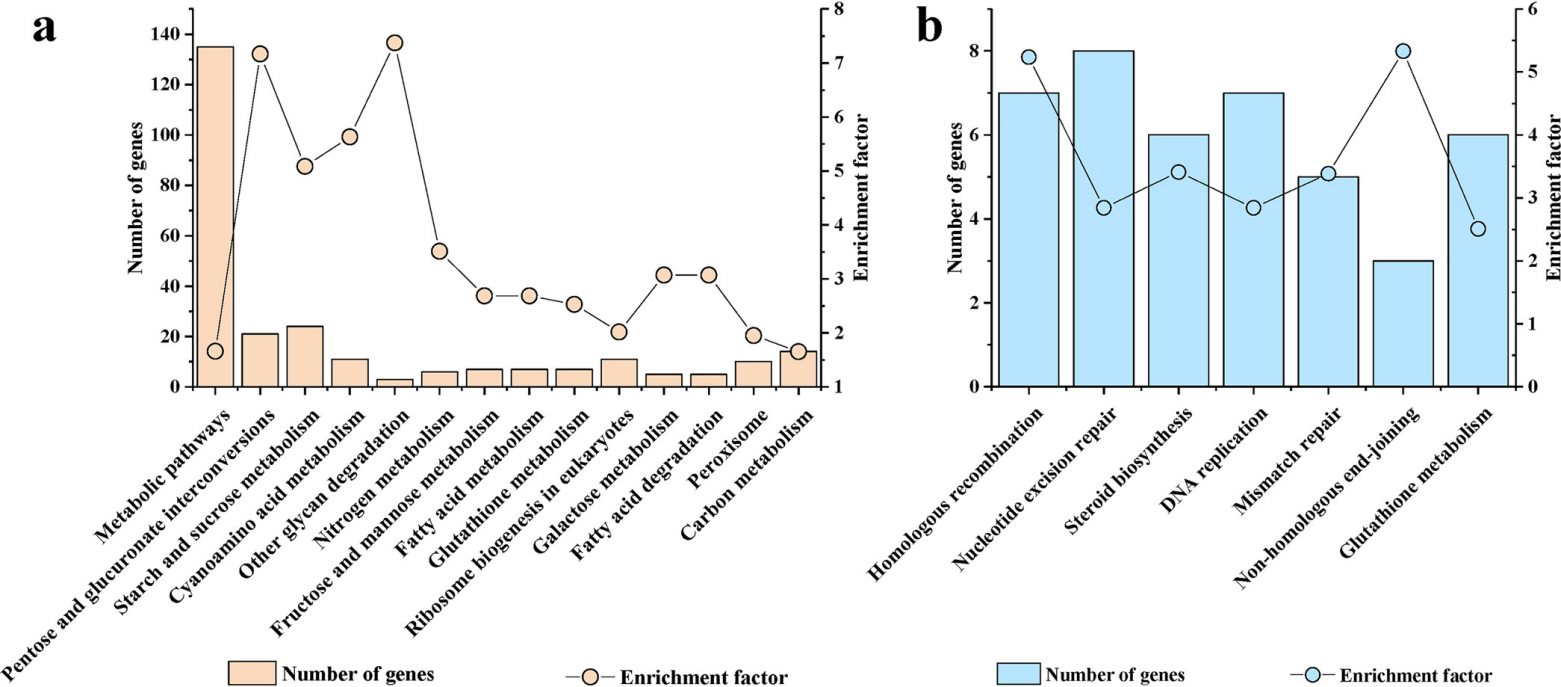
Kyoto Encyclopedia of Genes and Genomes (KEGG) enrichment analyses of upregulated and downregulated Sclerotinia sclerotiorum genes when strain DT-8 colonized rapeseed. (a) Enriched KEGG pathways of upregulated genes. (b) Enriched KEGG pathways of downregulated genes.

For upregulated *S. sclerotiorum* genes, the KEGG enrichment analysis showed similar results to the GO enrichment analysis, and there were 14 enriched pathways (Fig. 2a). In the KEGG pathway of starch and sucrose metabolism, 24 *S. sclerotiorum* genes were enriched. In addition to the genes linked to lignocellulose and pectin metabolism, there were five genes (*SS1G_01776*, *SS1G_08135*, *SS1G_10617*, *SS1G_13809*, and *SS1G_00249*) encoding glucoamylase or alpha-amylase (see Data Set S1, tab 8, in the supplemental material). This also suggested that starch might be an important nutrient for *S. sclerotiorum* DT-8.

For the downregulated *S. sclerotiorum* genes, the enriched pathways were also related to chromosomal duplication and included “homologous recombination,” “nucleotide excision repair,” “DNA replication,” “mismatch repair,” and “non-homologous end-joining.” Moreover, “steroid biosynthesis” was also enriched (Fig. 2b). As an important kind of steroid, ergosterol is involved in maintaining the integrity of cellular membranes in fungi (37). For *S. sclerotiorum*, sterol levels, especially the ergosterol content, also influence the infection (38). This result suggested that growth and steroid biosynthesis were decreased when *S. sclerotiorum* DT-8 colonized rapeseed.

### Differential expression of the ST genes of *S. sclerotiorum*.

**FIG 3:**
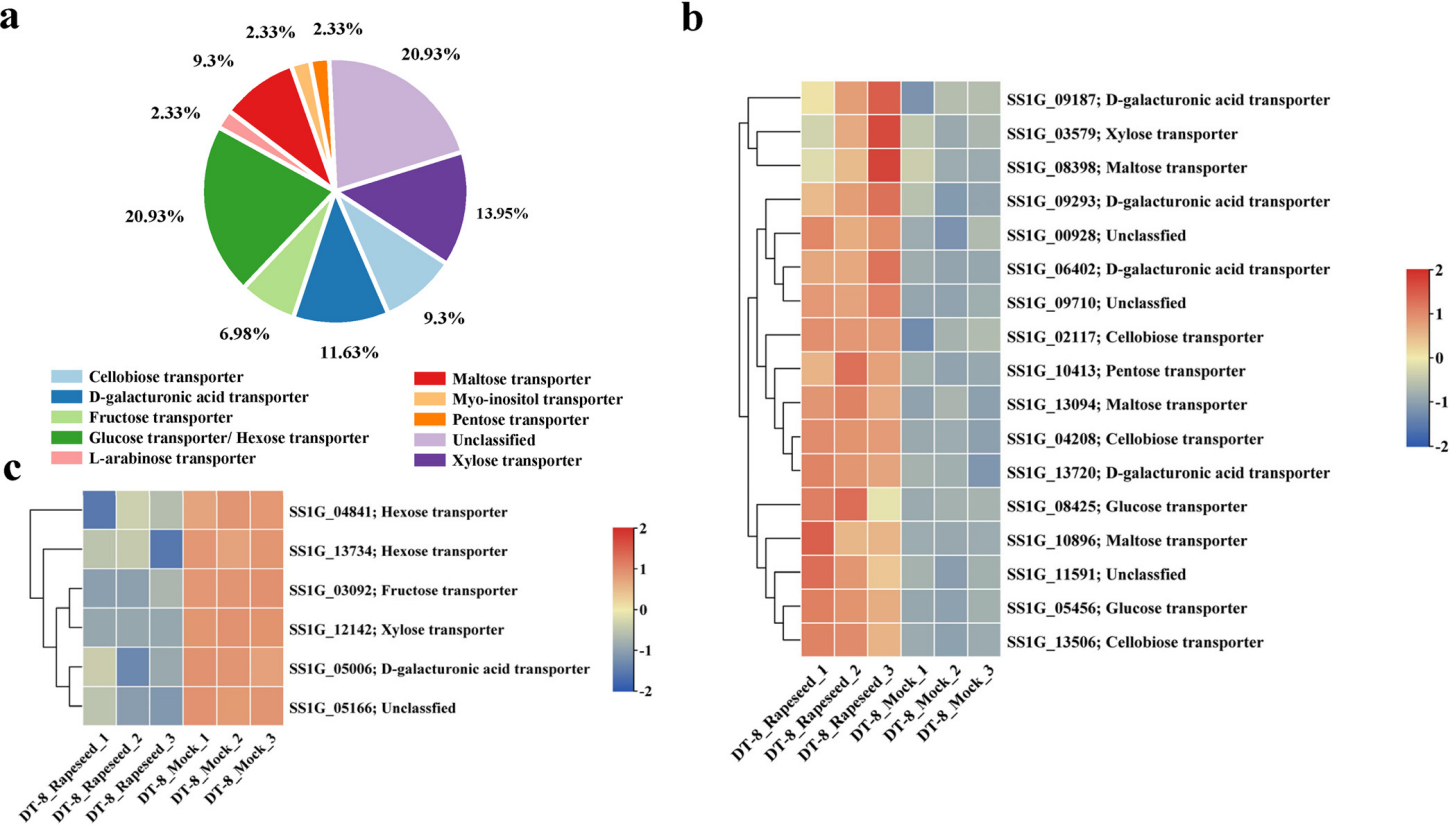
Expression profiles of sugar transporter genes of *S. sclerotiorum*. (a) Sugar transporters in *S. sclerotiorum*. (b) Expression profile of upregulated *S. sclerotiorum* sugar transporter genes. (c) Expression profile of downregulated *S. sclerotiorum* sugar transporter genes.

Sugar transporters constitute key components for carbon partitioning at the whole-plant level and in the interactions with fungi (34). We found 43 ST genes in the *S. sclerotiorum* genome (see Data Set S1, tab 9, in the supplemental material). According to the phylogenetic analysis, except for the unclassified ST, most ST genes encode glucose transporters/hexose transporters (Fig. 3a and Fig. S4 in the supplemental material). For the 17 upregulated *S. sclerotiorum* ST genes, there were three unclassified sugar transporter genes, three cellobiose transporter genes, four d-galacturonic acid transporter genes, three maltose transporter genes, one pentose transporter gene, one xylose transporter genes, and two glucose transporter genes (Fig. 3b). For the six downregulated *S. sclerotiorum* genes, there were two hexose transporter genes, one fructose transporter gene, one d-galacturonic acid transporter gene, one xylose transporter gene, and one unclassified sugar transporter gene (Fig. 3c). This result suggested that sugar transporters may play an important role when strain DT-8 colonizes rapeseed.

10.1128/mSystems.00814-21.4 FIG S4 Phylogenetic analysis of *S. sclerotiorum* sugar transporter genes. The phylogenetic tree was constructed in MEGA 7.0.26 by the neighbor-joining method with a bootstrap value of 1,000 replicates. Download FIG S4, TIF file, 2.9 MB. Copyright © 2021 Qu et al. 2021 Qu et al. https://creativecommons.org/licenses/by/4.0/ This content is distributed under the terms of the Creative Commons Attribution 4.0 International license.

### Expression profiles of secretory protein genes of *S. sclerotiorum*.

**FIG 4:**
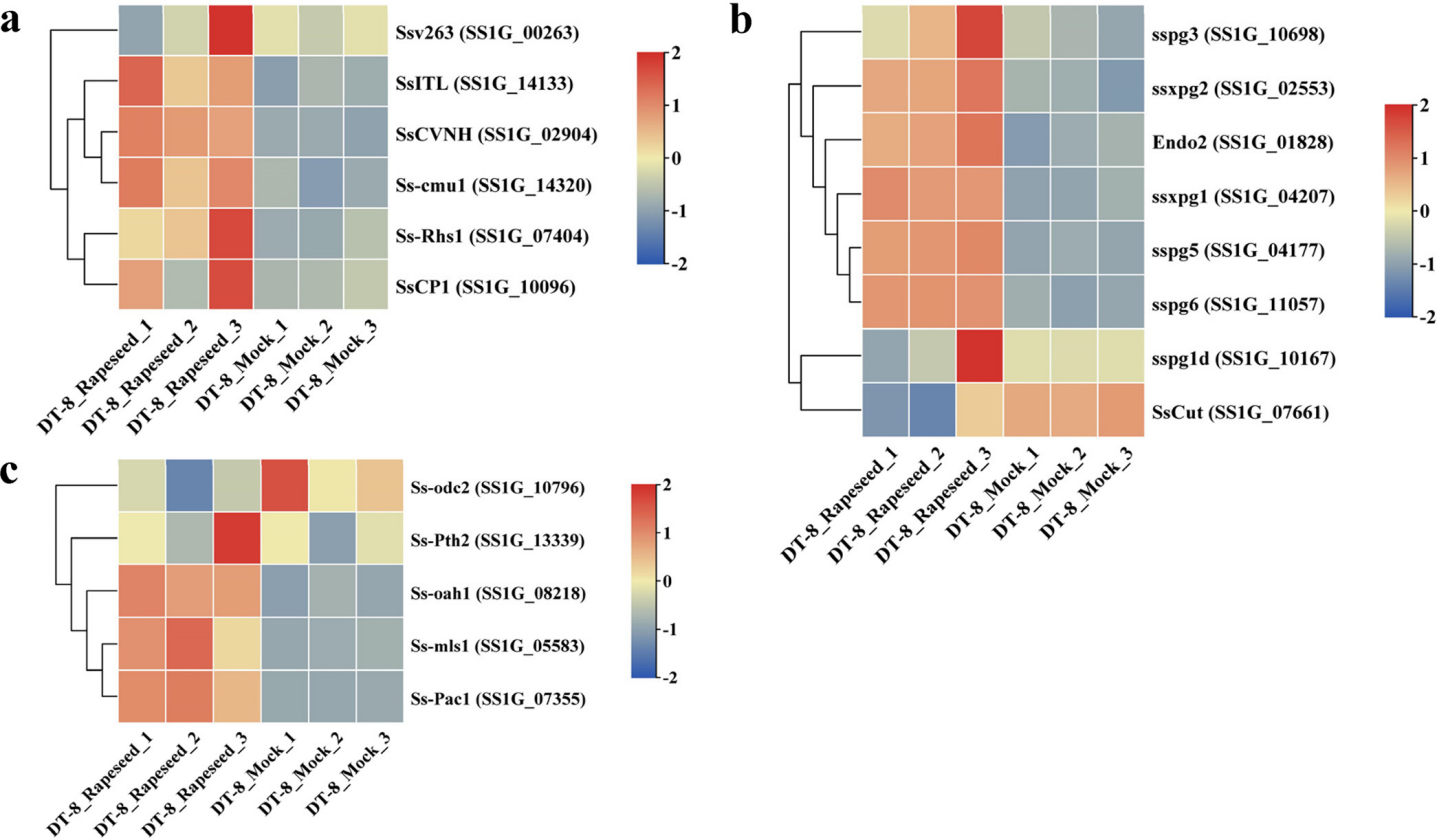
Expression profiles of *S. sclerotiorum* virulence factor genes. (a) Expression profiles of identified secretory protein (SP) genes in *S. sclerotiorum*. (b) Expression profiles of identified plant cell wall-degrading enzyme (PCWDE) genes in *S. sclerotiorum*. (c) Gene expression profiles of key genes of oxalic acid metabolism and regulation in *S. sclerotiorum*.

According to the prediction of Guyon et al. (39), the genome of *S. sclerotiorum* contains 486 genes encoding secretory proteins (SPs) expressed *in planta*. In our study, when colonizing rapeseed, 151 SP genes were upregulated and 25 were downregulated in *S. sclerotiorum* DT-8. Moreover, four upregulated SP genes were related to starch metabolism, e.g., the alpha amylase gene or glucoamylase gene (*SS1G_01776*, *SS1G_08135*, *SS1G_10617*, and *SS1G_13809*) (see Data Set S1, tab 10, in the supplemental material). These genes might play a crucial role in the carbon acquisition of strain DT-8. In *S. sclerotiorum*, 11 secreted proteins, including Ssv263, SsSSVP1, Ss-Caf1, SsCVNH, SsNACα, Ss-Bi1, SsPemG1, Ss-Rhs1, SsCP1, SsITL, and Ss-cmu1, have important impacts on the virulence of *S. sclerotiorum* (40). When colonizing rapeseed, *Ss-Rhs1*, *SsITL*, *Ss-cum1*, *SsCP1*, and *SsCVNH* were upregulated in strain DT-8 (Fig. 4a). These genes might help strain DT-8 eliminate the adverse effect of the activated defense response of rapeseed.

### Expression of plant cell wall-degrading enzyme genes of *S. sclerotiorum*.

Based on Lyu et al. (41), we studied the expression profiles of plant cell wall-degrading enzymes (PCWDE) genes of *S. sclerotiorum*. When strain DT-8 colonized rapeseed, 77 PCWDE genes were upregulated and seven were downregulated (see Fig. S5 in the supplemental material). Among the PCWDE genes identified previously (40), *sspg6*, *ssxpg1*, *Endo2*, *sspg5*, *ssxpg2*, and *sspg3* were upregulated in strain DT-8 (Fig. 4b). These upregulated PCWDE genes might promote the colonization of strain DT-8.

10.1128/mSystems.00814-21.5 FIG S5 Heatmap of differentially expressed plant cell wall-degrading enzyme (PCWDE) genes. Download FIG S5, TIF file, 2.9 MB. Copyright © 2021 Qu et al. 2021 Qu et al. https://creativecommons.org/licenses/by/4.0/ This content is distributed under the terms of the Creative Commons Attribution 4.0 International license.

### Expression profiles of oxalic acid metabolism and regulation genes.

For *S. sclerotiorum*, oxalic acid (OA) is a multifunctional molecule with a range of functions, such as the creation of a low-pH environment to facilitate PCWDEs, chelation of calcium to weaken the host cell wall structure, and reduction of host calcium toxicity, suppressing host defense responses (including the oxidative burst and callose deposition), and so on (42). When the hypovirulent strain DT-8 colonized rapeseed, the malate synthase gene *Ss-mls1*, the oxaloacetate acetylhydrolase gene *Ss-oah1*, and its positive transcription factor gene *Ss-PacI* were upregulated; moreover, the oxalate decarboxylase enzyme gene *Ssodc2* was downregulated (Fig. 4c). This result indicated that OA is also important for the colonization of DT-8 and might inhibit the activated defense response of rapeseed to favor the survival of DT-8. The low-pH environment might also aid the establishment of the symbiotic relationship between strain DT-8 and rapeseed.

### Expression profiles of other detoxification genes.

*S. sclerotiorum* is exposed to many toxic compounds produced by the host, such as isothiocyanates, host-derived reactive oxygen species (ROS), and phytoalexins. Correspondingly, *S. sclerotiorum* has a set of detoxification mechanisms. *SsSaxA* (*SS1G_12040*) encodes isothiocyanate hydrolase, which can degrade toxic isothiocyanates (43). *Ss-BGT1* (*SS1G_09997*) encodes a putative brassinin glucosyltransferase that detoxifies brassinin (44). In this study, *SsSaxA* and *Ss-BGT1* were upregulated in DT-8_Rapeseed (see Fig. S6 in the supplemental material). These genes were associated with the upregulated expression of indole-sulfur phytoalexin synthesis genes in rapeseed and contributed to the endogenous growth of strain DT-8. Three *S. sclerotiorum* genes (*SsCTR1* [*SS1G_05578*], *SsCCS* [*SS1G_00102*], and *SsATX1* [*SS1G_10888*]) are involved in fungal ROS detoxification by utilizing host-derived copper (45). Cu/Zn superoxide dismutase (SsSOD1 [SS1G_00699]), thioredoxin reductase (SsTrr1 [SS1G_05899]), survival factor 1 (SsSvf1 [SS1G_01919]), and type A catalase (SsCAT1 [SS1G_02784]) also play critical roles in the detoxification of ROS during host-pathogen interactions (38, 44, 46, 47). When strain DT-8 colonized rapeseed, the expression level of *SsCAT1* was higher in DT-8_Rapeseed than that in DT-8_Mock (see Fig. S6 in the supplemental material).

10.1128/mSystems.00814-21.6 FIG S6 Gene expression profiles of other detoxification genes of *S. sclerotiorum*. Download FIG S6, TIF file, 1.5 MB. Copyright © 2021 Qu et al. 2021 Qu et al. https://creativecommons.org/licenses/by/4.0/ This content is distributed under the terms of the Creative Commons Attribution 4.0 International license.

### Gene expression detection by quantitative reverse transcription-PCR.

To validate the results obtained in the dual-UMI RNA-seq experiments, quantitative reverse transcription-PCR (qRT-PCR) was used to analyze the relative expression of 13 *S. sclerotiorum* genes and 5 rapeseed genes. The results showed that the expression patterns of these representative genes were consistent with the transcriptome data (see Fig. S7 in the supplemental material). This result indicated that the transcriptome data were reliable.

10.1128/mSystems.00814-21.7 FIG S7 Expression of *S. sclerotiorum* and rapeseed genes detected by quantitative reverse transcription-PCR (qRT-PCR) and RNA-seq. Download FIG S7, TIF file, 1.1 MB. Copyright © 2021 Qu et al. 2021 Qu et al. https://creativecommons.org/licenses/by/4.0/ This content is distributed under the terms of the Creative Commons Attribution 4.0 International license.

## DISCUSSION

**FIG 5:**
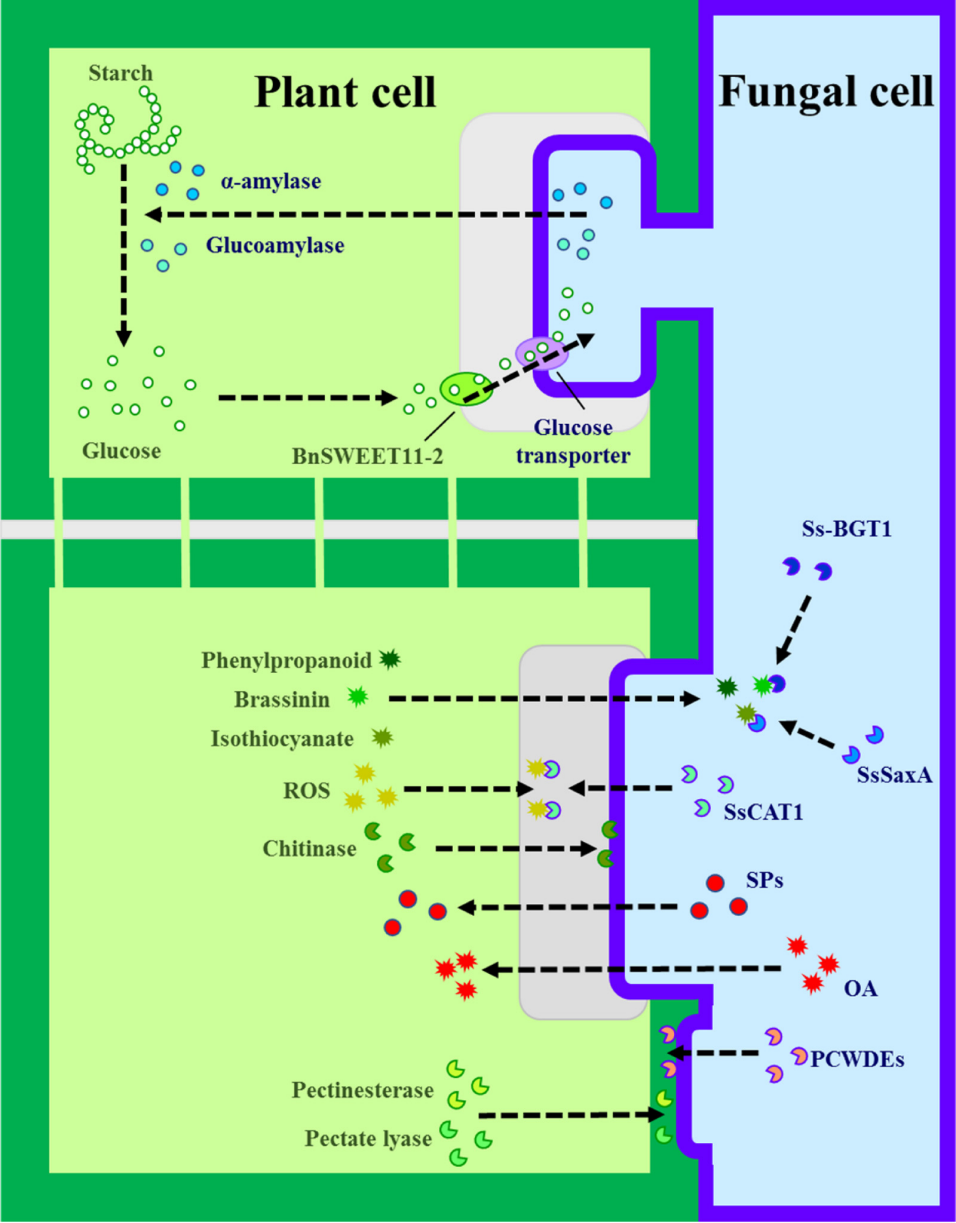
Symbiotic mechanisms between *S. sclerotiorum* DT-8 and rapeseed. BnSWEET11-2; *B. napus* sugars will eventually be exported transporter 11-2; ROS, reactive oxygen species; Ss-BGT1, *S. sclerotiorum* brassinin glucosyltransferase; SsSaxA, *S. sclerotiorum* isothiocyanate hydrolase; SsCAT1, *S. sclerotiorum* type A catalase; SPs, secretory proteins; OA, oxalic acid; PCWDEs, plant cell wall-degrading enzymes.

In this study, through dual-UMI RNA-seq, we compared *S. sclerotiorum* and rapeseed DEGs, when strains DT-8 colonized in the rapeseed and then resolved the symbiotic mechanisms between strain DT-8 and rapeseed (Fig. 5). For rapeseed, the upregulated expression of rapeseed pectinesterase and pectate lyase genes might promote the colonization of strain DT-8. At the same time, the activated defense response of rapeseed could not only increase the disease resistance but may also regulate the growth of strain DT-8. With the help of plant cell wall-degrading enzymes, oxalic acid, and secretory proteins, strain DT-8 could colonize rapeseed tissue, and with the action of *S. sclerotiorum* amylase/glucoamylase, sugar transporters, and rapeseed SWEET, strain DT-8 might use starch as a key nutrient. Moreover, the upregulation of detoxification genes in strain DT-8 was also conducive to its colonization.

Similarly to the results of Zhang et al. (27), in this study, many defense responses of rapeseed were activated after the colonization of strain DT-8, which enhanced the resistance of rapeseed and worsened the living environment of strain DT-8. This raises an interesting question of how strain DT-8 survives the activated defense responses of rapeseed. According to the RNA-seq data, an elaborately balanced antagonism also occurred in the interaction of strain DT-8 and rapeseed. With the action of OA, SPs, isothiocyanate hydrolase, and brassinin glucosyltransferase, strain DT-8 addresses the challenges brought by the activated defense responses of rapeseed. However, strain DT-8 could not completely inhibit the defense responses of rapeseed by its virulence factors; rapeseed also could not entirely prevent the growth of strain DT-8 by its defense responses. This trade-off is the cornerstone of the symbiosis between rapeseed and strain DT-8. Looked at from another aspect, the limited growth of strain DT-8 in rapeseed is also a key factor for its endophytic survival strategy.

Starch is the main form in which plants store carbohydrates (48). Endophytic fungi could produce starch-degrading enzymes and utilize starch as a source of nutrition *in vitro* (49). Choi et al. isolated 21 endophytic isolates from Brucea javanica, all of which produced amylase, and pointed out that starch might be consumed by the endophytes after the plant host dies (50). However, very little research has addressed starch utilization by endophytic fungi in living host plants. Isaeva et al. found that plant storage tissues that were rich in starch always contained many endophytic yeasts that were able to actively reproduce in these tissues without visible damage (51). In this study, we found that starch might be the carbon source for strain DT-8. When strain DT-8 colonized rapeseed, six *S. sclerotiorum* glucoamylase or alpha amylase genes (*SS1G_08135*, *SS1G_13809*, *SS1G_10617*, *SS1G_00249*, *SS1G_09392*, and *SS1G_01776*) were upregulated, of which four genes (*SS1G_01776*, *SS1G_08135*, *SS1G_10617*, and *SS1G_13809*) also encoded secretory proteins. Moreover, there were one upregulated rapeseed SWEET11 gene (*BnaA06g16330D*) and two upregulated *S. sclerotiorum* glucose transporter genes (*SS1G_05456*, *SS1G_08425*). For rice, OsSWEET11 could be induced by Xanthomonas oryzae and led to glucose efflux. Then, X. oryzae could take up glucose and multiply (52). Although the mechanism by which *S. sclerotiorum* secretory alpha amylase or glucoamylase enters rapeseed cells remains unclear, our results suggest that plant starch might be an important source of nutrients for strain DT-8. Moreover, the rapeseed sucrose transport protein SUC1 gene (*BnaC06g32880D*) was upregulated when strain DT-8 colonized rapeseed. In mycorrhizal roots, plant sucrose transporters play a significant role in carbohydrate segmentation between arbuscular mycorrhiza fungi (AMF) and plants (53). This result suggested that strain DT-8 might also utilize sucrose in the aerial parts of rapeseed, as AMF do in plant roots.

Oxalic acid is the most ubiquitous and common low-molecular-weight organic acid produced by living organisms. For fungi, OA plays important roles in pathogenicity, wood degradation, mineral weathering, nutrient acquisition, and metal tolerance; for plants, OA and oxalate play major roles in calcium regulation, ionic balance, heavy metal detoxification, and plant defense against herbivores (54). Endophytic fungi are also the producers of OA (55). Moreover, colonization by endophytic fungi could change the concentration of OA in plants. In mycorrhizal maize with the AMF Glomus mosseae, the dominant organic acid was OA, while in nonmycorrhizal maize plants, it was succinic acid (56). In our study, when strain DT-8 colonized rapeseed, the biosynthesis genes of OA were upregulated. On one hand, OA secreted by strain DT-8 might inhibit the activated defense response of rapeseed; on the other hand, the low-pH environment created by OA might also be beneficial to the interaction of strain DT-8 and rapeseed.

Secreted proteins have been proven to promote colonization by manipulating host defense and reprogramming plant metabolism during symbiosis (57). The effector genes of the ectomycorrhizal fungus Laccaria bicolor, *MiSSP7* and *MiSSP8*, were highly upregulated during symbiosis with poplar roots, and with reduced expression of *MiSSP7* or *MiSSP8*, L. bicolor could not establish symbiosis with Populus (58, 59). The transcriptome when the endophytic fungus Epichloë festucae infected Festuca rubra showed that 19 abundantly expressed fungal secreted small cysteine-rich proteins might have a role in the endophyte-host symbiosis (60). RNA-seq showed that 11 secreted protein genes were upregulated when the AMF Rhizophagus irregularis was in a state of symbiosis, and colonization by the top upregulated *SIS1* host-induced gene silencing (HIGS)-mediated knockdown mutant was suppressed in the hairy roots of Medicago truncatula (61). For *S. sclerotiorum*, secreted proteins have diverse roles in both development and virulence (62). In our research, 151 secreted protein genes were upregulated when strain DT-8 colonized rapeseed. These genes might play important roles in colonization by strain DT-8 and should be further investigated in the future.

Plant pectate lyase results in plant cell wall disassembly and plant tissue softening (63) and is an important factor in the interaction between microorganisms and plants. Transcriptome analysis showed that many Lotus japonicus pectate lyase genes were induced during the early stages of symbiosis with Mesorhizobium loti (64). Xie et al. found that the L. japonicus nodulation pectate lyase gene (*LjNPL*) was induced in roots and root hairs by rhizobial nodulation (Nod) factors of M. loti, and *LjNPL* mutants produced uninfected nodules (65). Mutants of the pectate lyase-like protein gene (*PMR6*) of Arabidopsis showed increased resistance to powdery mildew, and *pmr6*-mediated resistance was not dependent on signaling through the salicylic acid or jasmonic acid/ethylene pathways (66). This result indicated that the expression of plant pectate lyases might be beneficial to the colonization of microorganisms. When strain DT-8 colonized rapeseed, we also found that rapeseed pectinesterase and pectate lyase genes were upregulated. These genes might be induced by strain DT-8 and be beneficial for the colonization of strain DT-8.

As a virus-mediated endophytic fungus, *S. sclerotiorum* DT-8 could colonize in rapeseed and showed a beneficial symbiotic character with rapeseed. In this research, through the transcriptome data, we found that the key lifestyle factors were the changes in the mode of nutrient acquisition and the balance of antagonism between the host and the endophyte. However, this research still has limitations. First, this research was performed in an artificial environment that was quite different from the natural ecosystems. Second, the sample size was also limited in our research. Despite such flaws, our study still sheds light on the possible targets for future investigations of the mechanisms underlying the symbiosis of virus-mediated endophytic fungi and their hosts.

## MATERIALS AND METHODS

### Fungus materials.

The hypovirulent *S. sclerotiorum* strain DT-8 infected with SsHADV-1 was originally isolated from a sclerotium formed on a diseased stem of rapeseed (26). Strain DT-8 was subcultured on potato dextrose agar (PDA) plates at 20°C.

### Rapeseed biopriming with *S. sclerotiorum* DT-8.

Rapeseeds (Brassica napus cv. Huashuang 4) were surface sterilized by 2% sodium hypochlorite solution for 5 min, followed by three successive thorough rinses with sterilized distilled water (SDW). Strain DT-8 was cultured in a shaken flask in potato dextrose broth (PDB) medium for 5 days at 200 rpm and 20°C. Then, the hyphal fragment suspension was diluted to 2.0 optical density at 600 nm (OD_600_) units with SDW for biopriming. The sterilized rapeseeds were primed with strain DT-8 by mixing 5 g of seeds with 10 ml of mycelial suspension. After 18 h of treatment at 20°C, the seeds were air-dried to a constant weight. Nonbioprimed seeds soaked with SDW at 20°C for 18 h were used as controls. All bioprimed and nonbioprimed seeds were planted on starch-less MSA medium in bottles for follow-up experiments. All plants were maintained in the greenhouse at 20°C with 12 h light and 12 h dark for 14 days.

### Sample collection, RNA extraction, library preparation, and sequencing.

The experimental scheme of dual-UMI RNA-seq is shown in Fig. 1. For the DT-8 treatment group, 20 hypocotyls of 14-day-old bioprimed rapeseed plants were collected to explore *S. sclerotiorum* gene expression (DT-8_Rapeseed) and rapeseed gene expression (Rapeseed_DT-8). The mycelia of strain DT-8 growing on PDA medium for 3 days were used as a control group (DT-8_Mock). Twenty hypocotyls of 14-day-old noninoculated rapeseed plants were also collected as the control for rapeseed gene expression analysis (Rapeseed_Mock). Each treatment had three biological replicates.

Total RNA of all samples was extracted using TRIzol (Invitrogen) following the manufacturer’s protocol (67). DNA digestion was carried out using DNase I. RNA quality was determined by examining *A*_260_/*A*_280_ with a NanoDrop One C spectrophotometer (Thermo Fisher Scientific, Inc.). RNA integrity was confirmed by 1.5% agarose gel electrophoresis. Qualified RNAs were finally quantified by Qubit 3.0 with a Qubit RNA broad-range assay kit (Life Technologies). Two micrograms of total RNA were used for stranded RNA sequencing library preparation using the KC-Digital stranded mRNA library prep kit for Illumina (catalog no. DR08502; Wuhan Seqhealth Technology Co., Ltd., People’s Republic of China) following the manufacturer’s instructions. The kit eliminated duplication bias during PCR and sequencing steps by using a unique molecular identifier (UMI) of 8 random bases to label the preamplified cDNA molecules. The library products corresponding to 200 to 500 bp were enriched, quantified, and finally sequenced on a HiSeq X Ten sequencer (Illumina).

### RNA-Seq data analysis.

Raw sequencing data were first filtered by Trimmomatic (version 0.36) (68); then, low-quality reads were discarded and the reads contaminated with adaptor sequences were trimmed. Clean reads were further treated with KC-UID (the official analysis software of Seqhealth Technology Co., Ltd., used to process reads of the UMI RNA-seq library; https://github.com/KC-UID/KC-UID) to eliminate duplication bias introduced during library preparation and sequencing. In brief, clean reads were first clustered according to the UMI sequences; reads with the same UMI sequence were grouped into the same cluster. Reads in the same cluster were compared to each other by pairwise alignment, and then reads with a sequence identity of over 95% were extracted to a new subcluster. After all of the subclusters were generated, multiple-sequence alignments were performed to get one consensus sequence for each subcluster. After these steps, all errors and biases introduced by PCR amplification or sequencing were eliminated.

The deduplicated consensus sequences were used for standard RNA-seq analysis. They were mapped to the reference genomes of *S. sclerotiorum* strain 1980 UF-70 (NCBI Genome assembly accession no. ASM14694v2) (69) and rapeseed (from the Genoscope Genome Database; http://www.genoscope.cns.fr/brassicanapus/data/) (70) using Spliced Transcripts Alignment to a Reference (STAR) software (version 2.5.3a) (71) with default parameters. Reads mapped to the exon regions of each gene were counted by *featureCounts* (72). Differentially expressed genes (DEGs) between groups were identified using the *edgeR* package (73) and were filtered using a threshold of false-discovery rate (FDR of) <0.05 and an absolute log_2_ fold change (log_2_FC) of >1. To avoid the noise signals from high-throughput sequencing, only genes detected in at least three biological replicates of one condition, above the detection threshold of 1 count per million (31), were used in this analysis. The principal-component analysis (PCA) was performed on the expression data using the “prcomp” function of R (version R x64 3.5.0; R Core Team, Vienna, Austria). Genes were annotated based on BLAST search results (E value, <10^−5^) against four public databases comprising the Kyoto Encyclopedia of Genes and Genomes (KEGG) (http://www.genome.jp/kegg/), UniProtKB/Swiss-Prot (http://www.uniprot.org), Pfam (http://pfam.xfam.org/), and InterPro (http://www.ebi.ac.uk/interpro/) databases. The functional annotation of gene ontology (GO) terms was analyzed by BLAST2GO. GO enrichment analysis was performed using Biological Directed acyclic graphs Gene Ontology (BiNGO) 3.0.3 tool (74) with FDR < 0.05, and we paid more attention to the GO terms which were end nodes in the directed acyclic graphs constructed by BiNGO (75). KEGG enrichment was conducted using the *clusterProfiler* package (76), and the threshold was set as a *P* value of <0.05. For the phylogenetic analysis, alignments were performed by Clustal W 2.0 (77) and phylogenetic trees were constructed in MEGA 7.0.26 (78) by the neighbor-joining method with a bootstrap value of 1,000 replicates.

### Quantitative real-time RT-PCR analysis.

Quantitative real-time RT-PCR (qRT-PCR) analysis for validating the different expression data was performed independently under the same conditions described above. First-strand cDNA was synthesized with an oligo(dT) primer using cDNA Synthesis SuperMix (TransGen Biotech, China). Quantitative real-time RT-PCR was carried out in a CFX96 real-time PCR detection system (Bio-Rad) with iTaq Universal SYBR Green Super Mix (Bio-Rad). PCR amplification was performed under the following conditions: 95°C for 3 min, followed by 55 cycles of 95°C for 15 s, 56°C for 15 s, and 72°C for 20 s. Melt curve profiles were analyzed for each gene tested at the end of each PCR. The ubiquitin genes of *S. sclerotiorum* (*SS1G_11035*) and B. napus (*UBC21*) served as the internal reference genes (79, 80). Primers for the target genes were designed using Beacon Designer V7.92 and are listed in Data Set S1, tab 11.

### Data availability.

All raw data from dual-UMI RNA-seq are available at the Sequence Read Archive (identifiers SRP268537 and SRP268377).
