# Supplementary figures and images for: Exploring the Symbiotic Mechanism of a Virus-Mediated Endophytic Fungus in Its Host by Dual Unique Molecular Identifier–RNA Sequencing
Source: mSystems. 2021 Sep 14;6(5):e00814-21. doi: 10.1128/mSystems.00814-21 (PMC8547468; doi:10.1128/mSystems.00814-21)

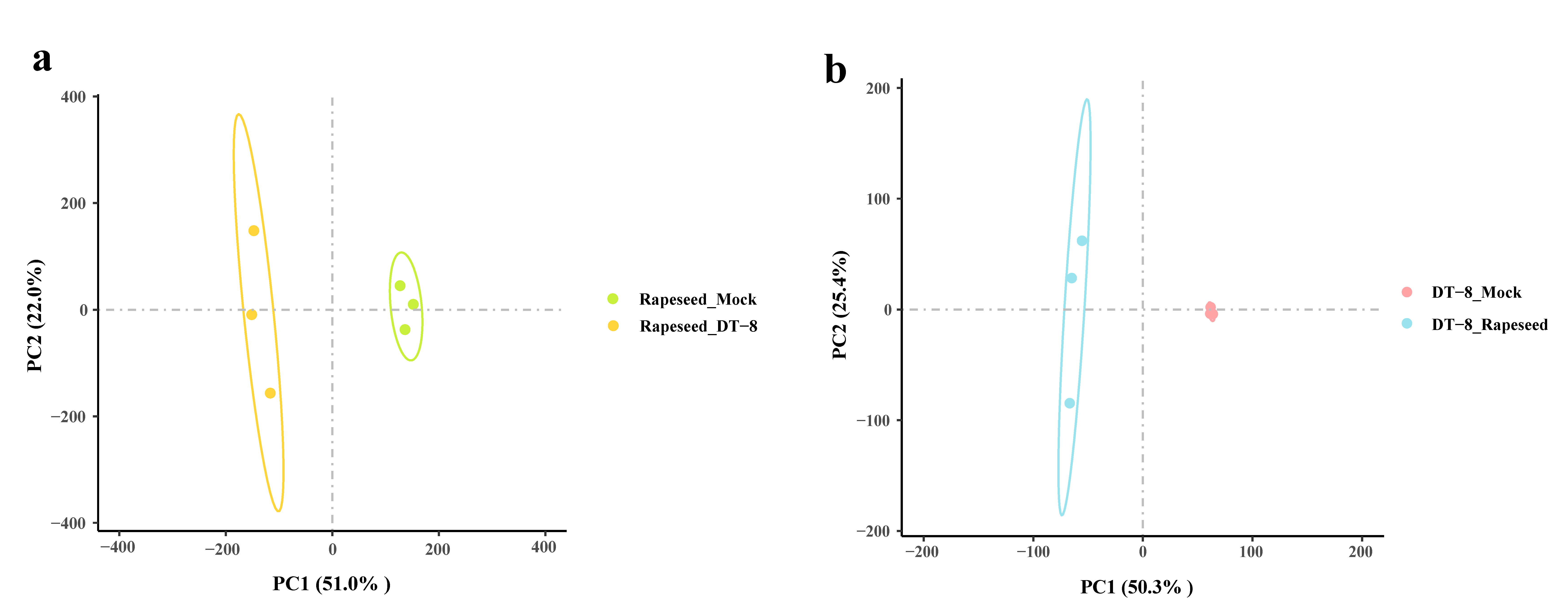

Supplement: FIG S1 [file msystems.00814-21-sf001.tif]

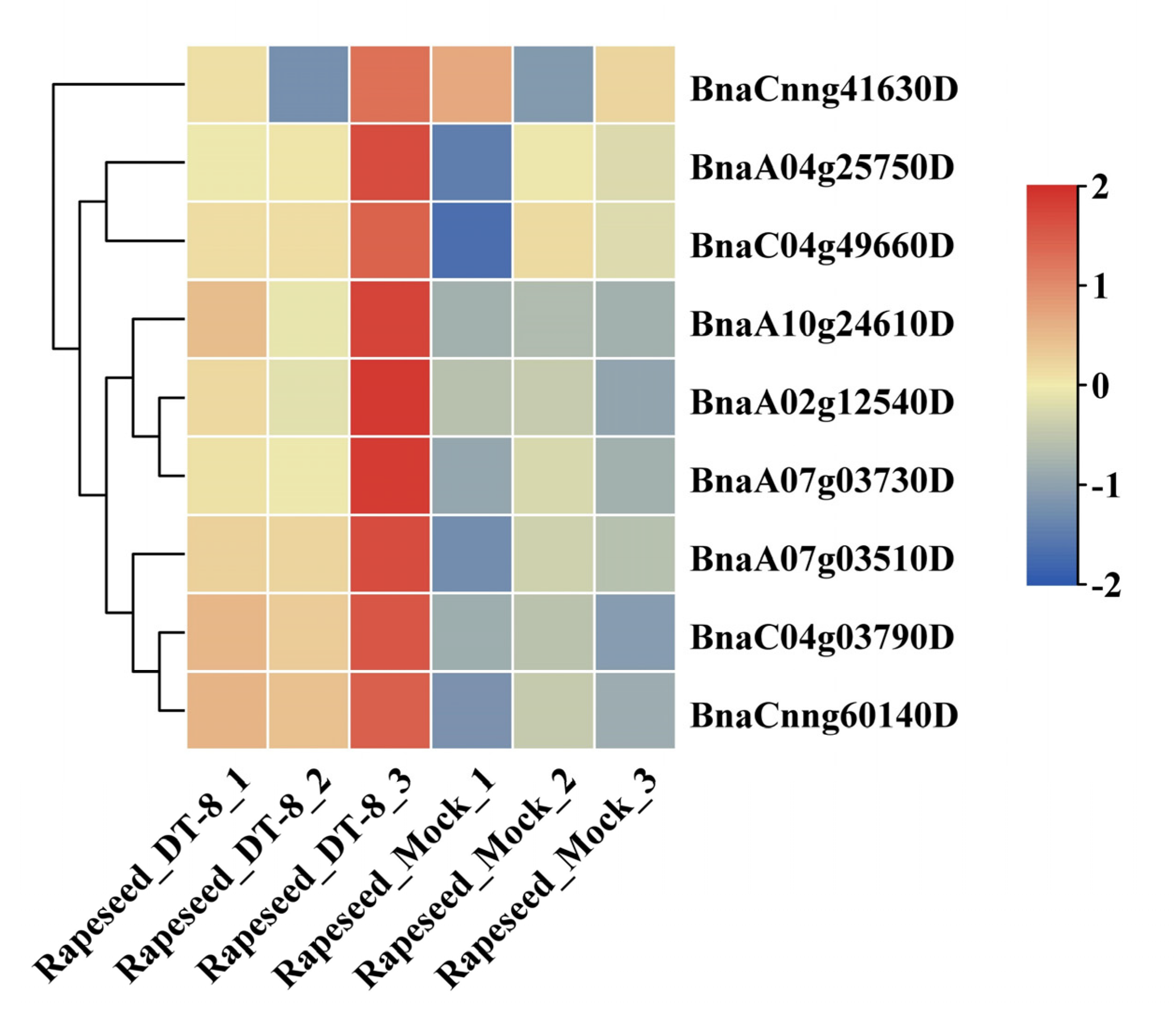

Supplement: FIG S2 [file msystems.00814-21-sf002.tif]

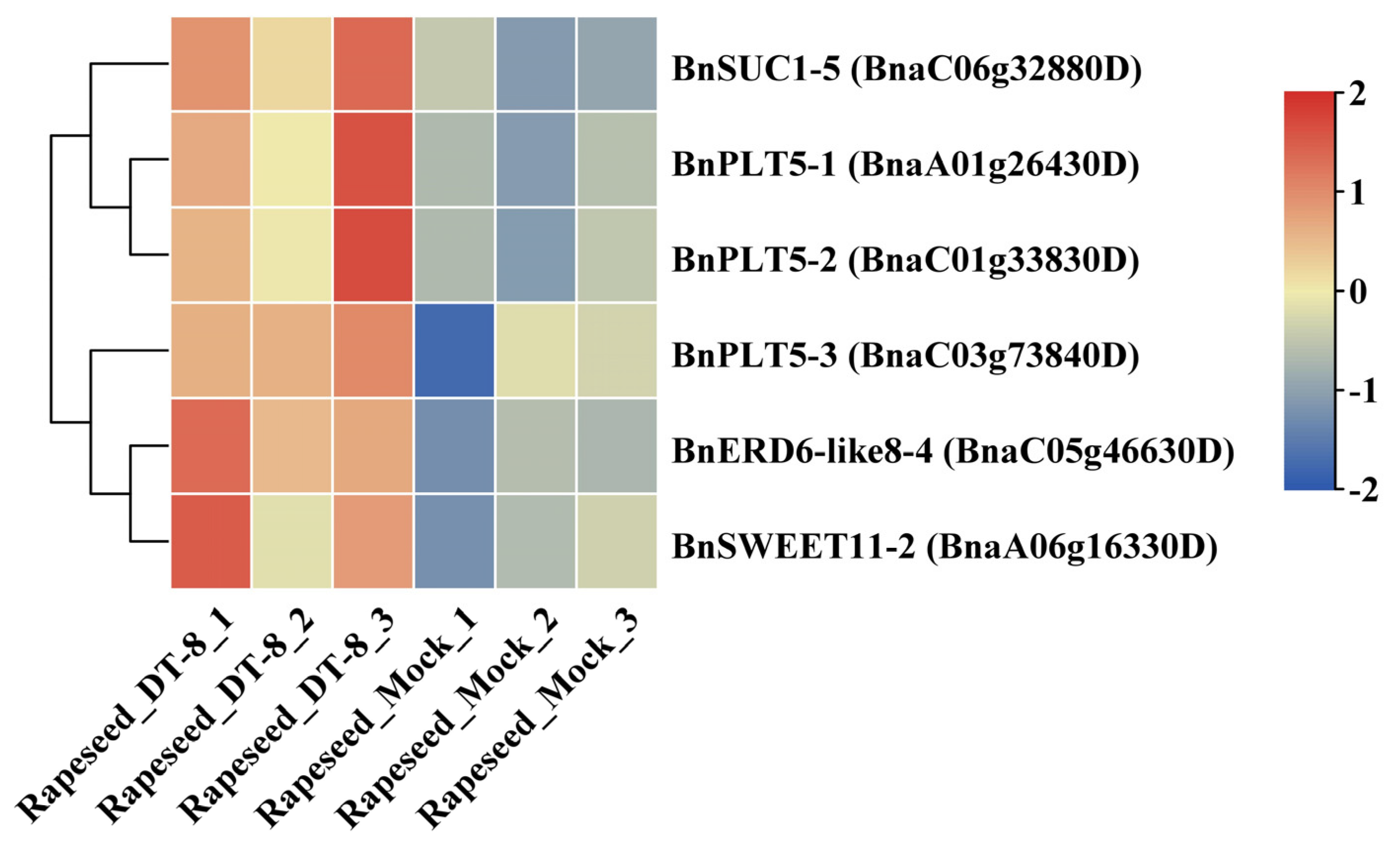

Supplement: FIG S3 [file msystems.00814-21-sf003.tif]

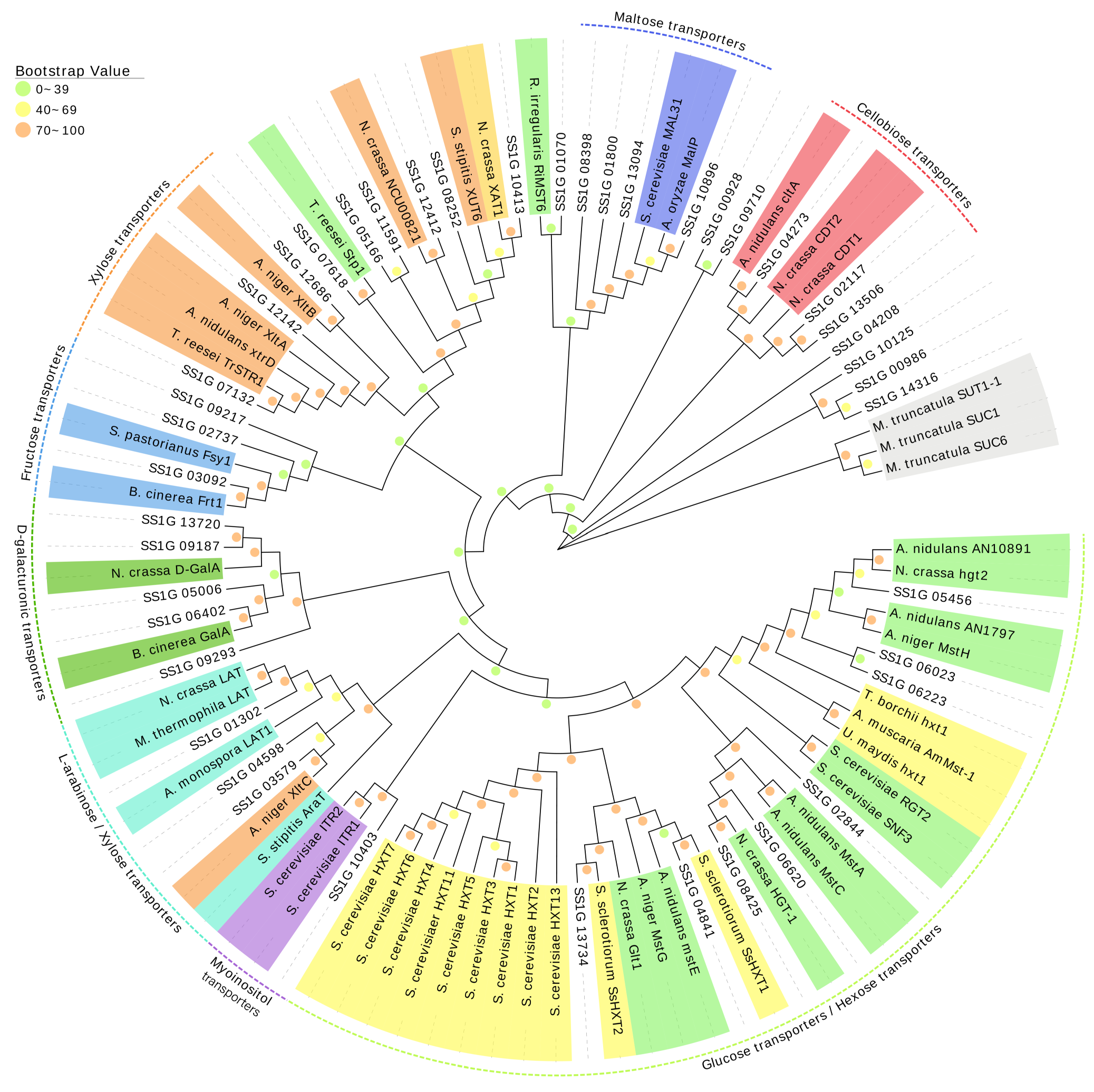

Supplement: FIG S4 [file msystems.00814-21-sf004.tif]

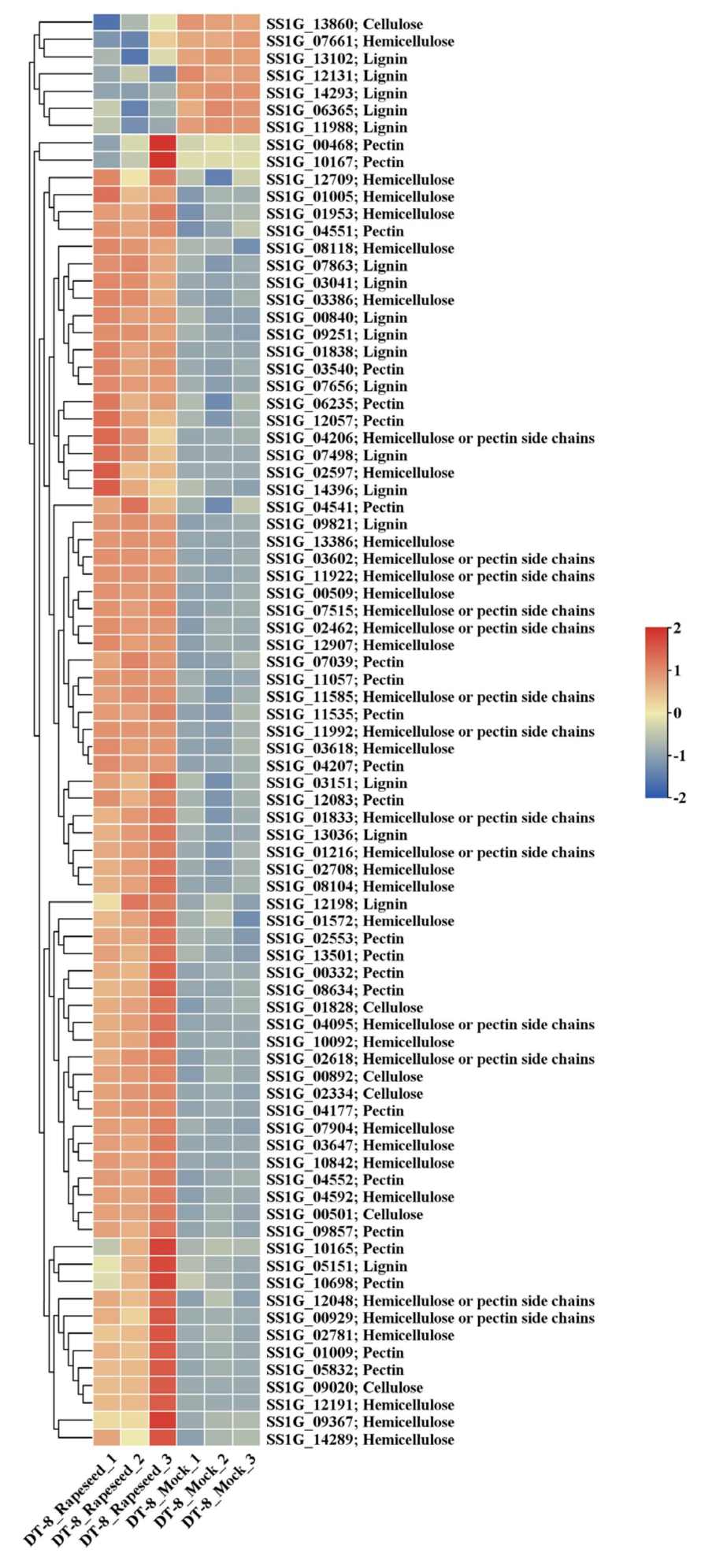

Supplement: FIG S5 [file msystems.00814-21-sf005.tif]

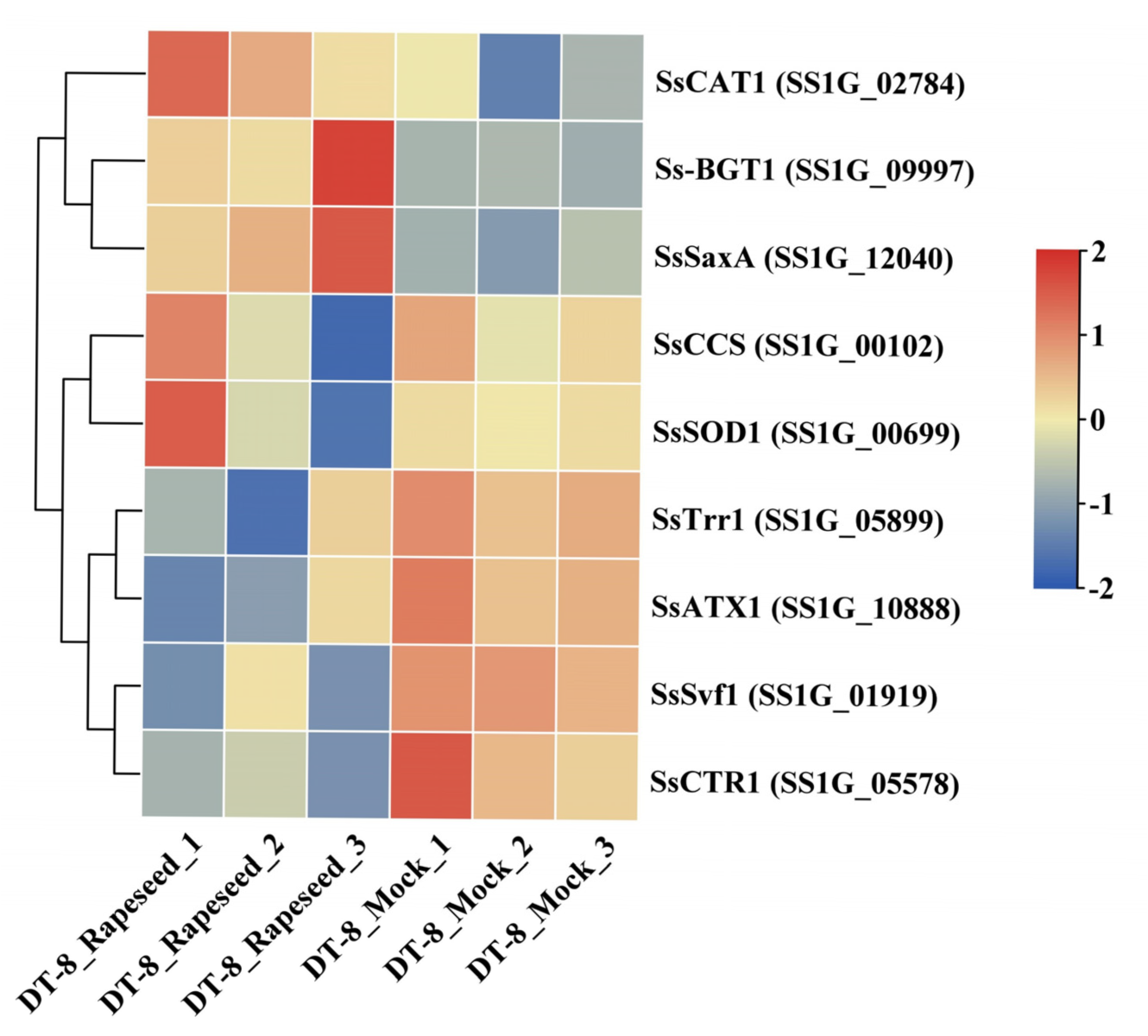

Supplement: FIG S6 [file msystems.00814-21-sf006.tif]

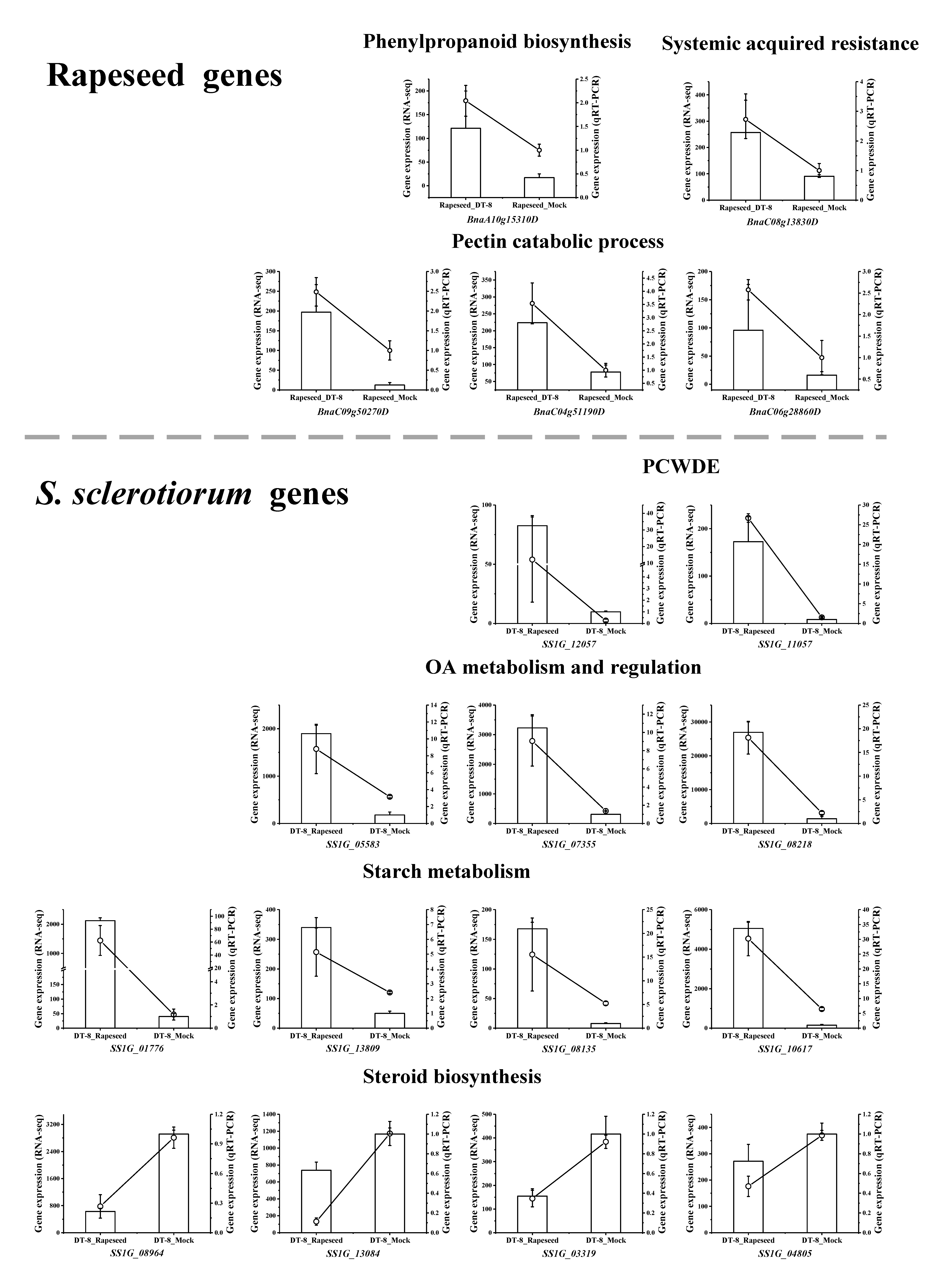

Supplement: FIG S7 [file msystems.00814-21-sf007.tif]
